# Supplementary figures and images for: Refining the root-associated microbial consortia for enhanced biocontrol of the root-rot pathogen of corn
Source: Front Microbiol. 2026 Apr 10;17:1714069. doi: 10.3389/fmicb.2026.1714069 (PMC13106159; doi:10.3389/fmicb.2026.1714069)

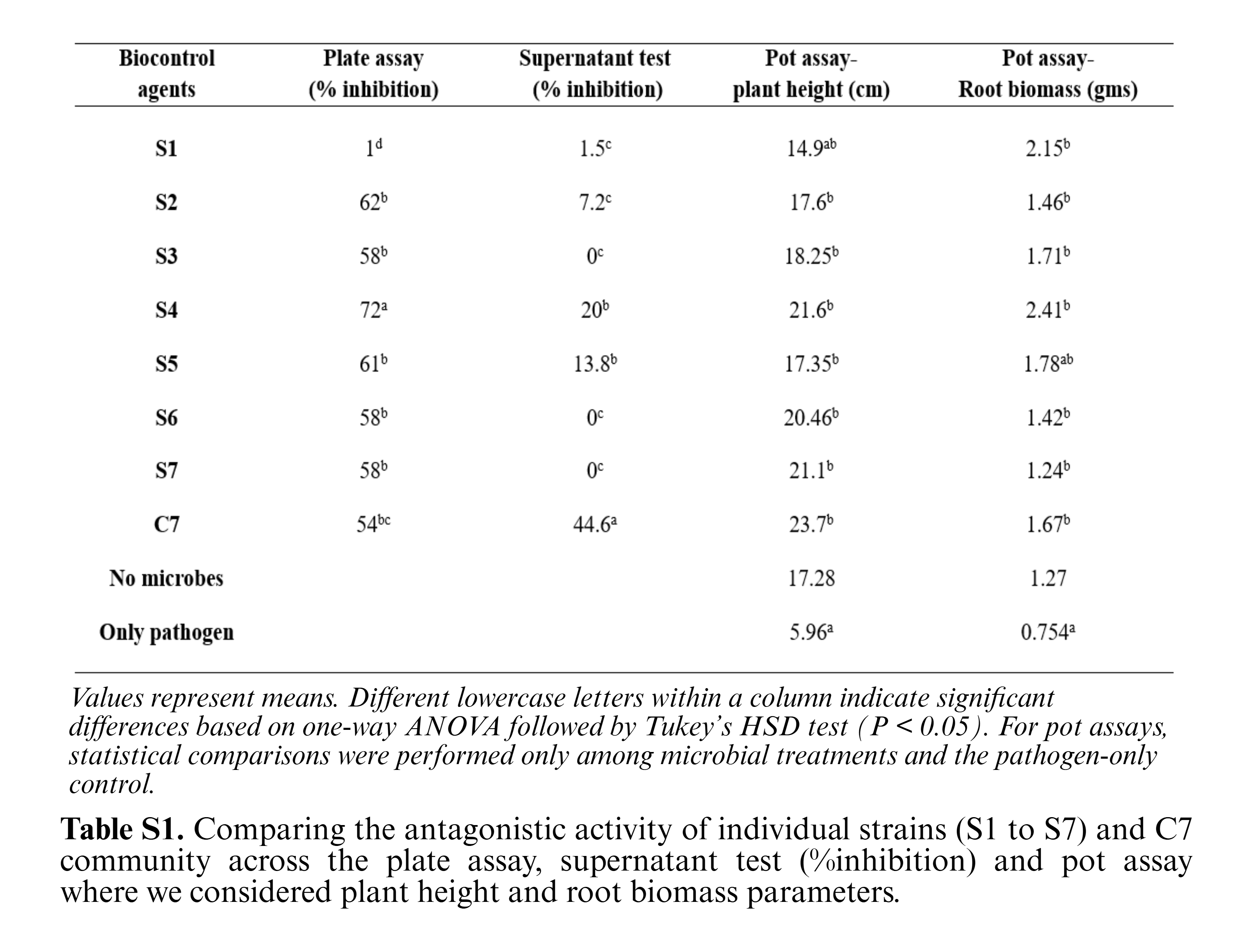

Supplement: Supplementary file 1 [file Supplementary_file_1.png]

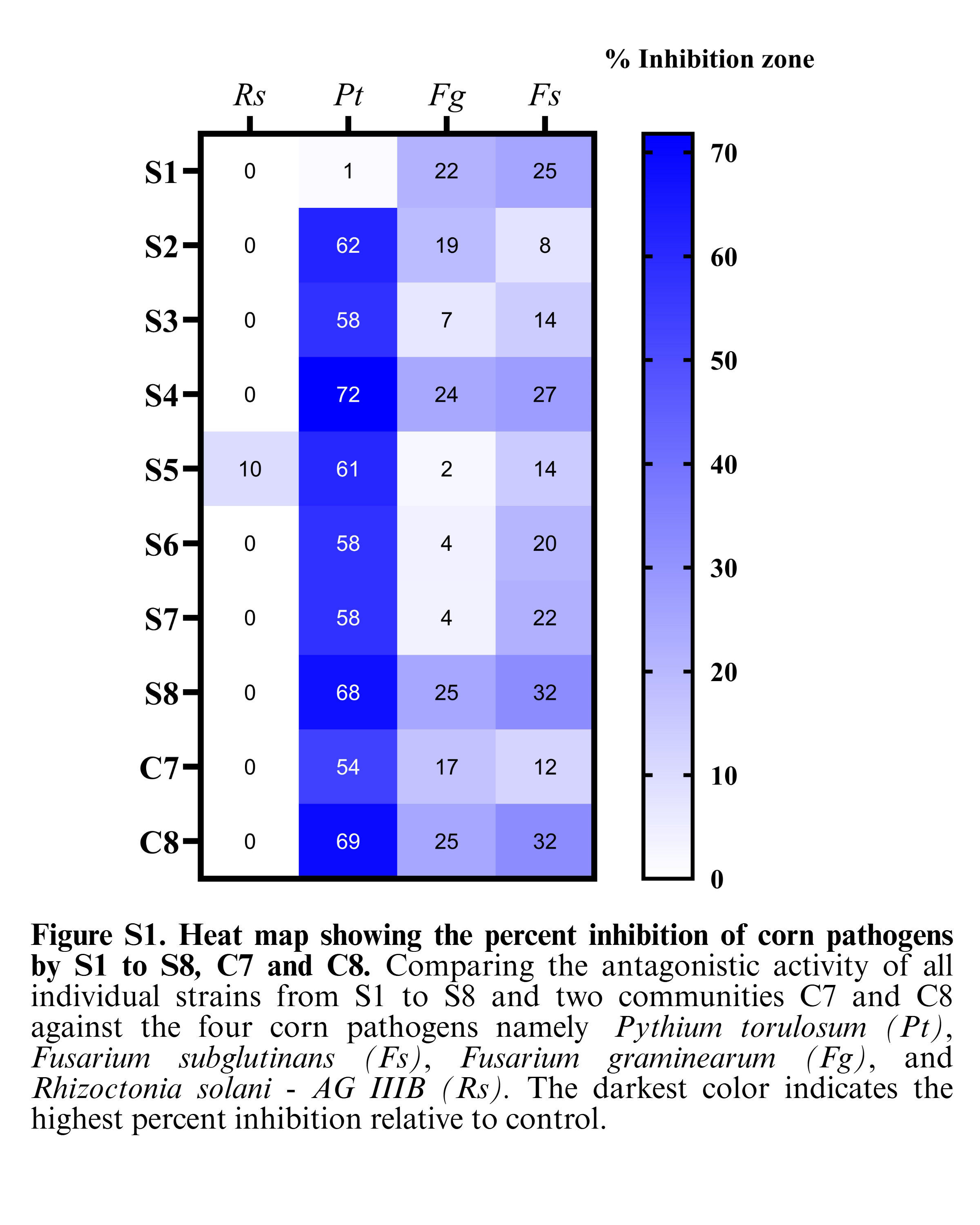

Supplement: Supplementary file 2 [file Image_1.png]

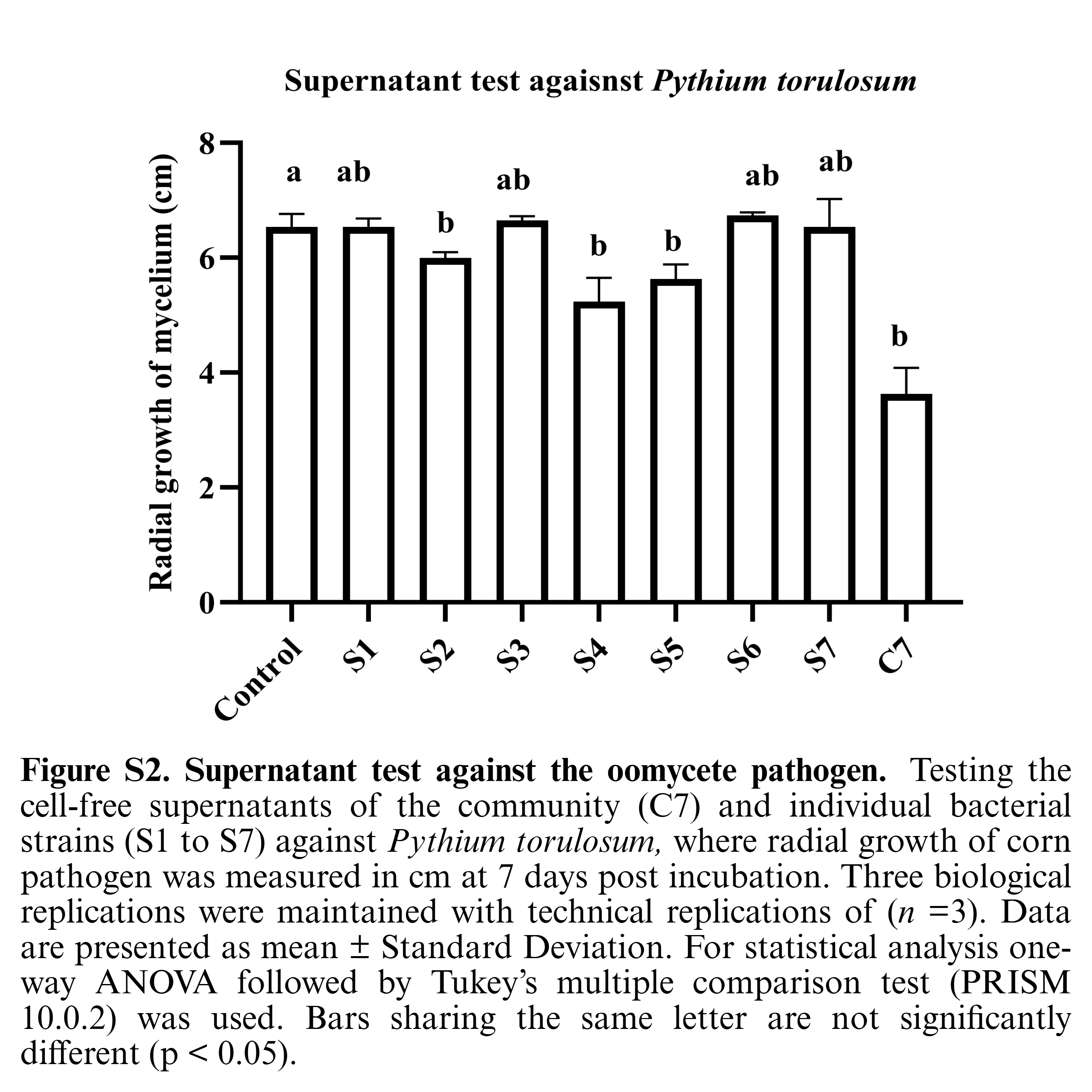

Supplement: Supplementary file 3 [file Image_2.jpeg]
